# Correlation between superoxide dismutase 1 and 2 polymorphisms and susceptibility to oral squamous cell carcinoma

**DOI:** 10.3892/etm.2013.1375

**Published:** 2013-10-31

**Authors:** YING LIU, LAGABAIYILA ZHA, BO LI, LOUQIANG ZHANG, TAO YU, LONGJIANG LI

**Affiliations:** 1 State Key Laboratory of Oral Diseases, West China Hospital of Stomatology, Sichuan University, Chengdu, Sichuan, P.R. China; 2 Department of Head and Neck Oncology Surgery, West China Hospital of Stomatology, Sichuan University, Chengdu, Sichuan, P.R. China; 3 Department of Forensic Science, School of Basic Medical Sciences, Central South University, Changsha, Hunan, P.R. China; 4 Department of Head and Neck Oncology, Sichuan Cancer Hospital, Chengdu, Sichuan, P.R. China

**Keywords:** superoxide dismutase 1, mitochondrial superoxide dismutase 2, oral squamous cell carcinoma, gene polymorphisms

## Abstract

Oxidative stress is significant in numerous types of cancer. Tobacco smoke, an important risk factor for oral squamous cell carcinoma (OSCC), is able to generate reactive oxygen species (ROS) and cause oxidative DNA damage. Superoxide dismutase (SOD) is an endogenous antioxidant enzyme that is critical in limiting the oxidative burden effectively. The purpose of this study was to investigate the effects of the mitochondrial SOD2 and Cu/Zn enzyme SOD1 gene polymorphisms on the susceptibility to and clinicopathological characteristics of OSCC, as well as the synergistic effect between these gene polymorphisms and the well-known risk factor of tobacco consumption. Patients with clinically diagnosed OSCC (n=362) and healthy normal individuals (n=358) were investigated for four single nucleotide polymorphisms (SNPs; rs4880, rs5746136, rs1804450 and rs11556620) by polymerase chain reaction-restriction fragment length polymorphism and DNA sequencing methods. Following adjustment for other confounders, no significant difference was observed in the rs5746136 SOD2 SNPs between the patients and controls. However, the incidence of the CT genotype of SOD2 SNP rs4880 was higher in the patients than in normal subjects in the additive model [CT vs. TT; P=0.045; adjusted odds ratio (AOR)=1.484; 95% confidence interval (CI), 1.009–2.182] and in the dominant model (CT/CC vs. TT; P=0.022; AOR=1.559; 95% CI, 1.067–2.278). For those who smoked, the incidence of the CT genotype of rs4880 increased markedly in the patients compared with the controls in the additive model (CT vs. TT; P=0.003; AOR=2.325; 95% CI, 1.330–4.064) and in the dominant model (CT/CC vs. TT; P=0.001; AOR=2.448; 95% CI, 1.417–4.230). For SOD1, polymorphisms at rs1804450 and rs11556620 were not present in any of the OSCC or control subjects. The results suggest that SOD2 rs4880 may be involved in the tumorigenesis of OSCC and may be useful as a genetic susceptibility marker for OSCC.

## Introduction

Oral squamous cell carcinoma (OSCC), the sixth most common type of cancer in the world, is a major cause of morbidity and mortality and >31,000 patients are newly diagnosed with OSCC in the USA annually (1). It is one of the most prevalent cancers in the human male population worldwide, with approximately two-thirds of all cases occurring in developing countries (2). Despite substantial effort and novel therapeutic developments, the five-year survival rate for OSCC has not appreciably improved over the last two decades (1,3). In China, the risk of morbidity due to OSCC is increasing, and the patients tend to be increasingly younger (4). OSCC is therefore a significant public health threat throughout the world (5).

It is widely accepted that the major risk factor for OSCC is chronic exposure of the oral epithelia to tobacco smoke and alcohol (6,7). Tobacco smoke produces a variety of free radicals, reactive oxygen species (ROS) and reactive nitrogen species (RNS), which cause oxidative damage and oxidative stress through the production of superoxide, hydrogen peroxide and nitric oxide in cells (8). Oxidative stress is significantly involved in several pathological conditions, including cardiovascular disease, neurological disorders, aging and cancer (8,9). Interactions between ROS and RNS are a crucial determinant of the etiology of OSCC, and conversely, antioxidants protect against the cellular and molecular damage caused by these interactions (10,11). Lin *et al* reported that cigarette smoking, alcohol consumption and betel quid chewing have all been identified as significant causes of oral carcinogenesis, primarily due to their ability to generate ROS and cause oxidative DNA damage (12).

Superoxide dismutases (SODs) are enzymes that catalyze the dismutation of superoxide into oxygen and hydrogen peroxide (13). There are three major families of superoxide dismutase, depending on the metal cofactor: Cu/Zn (SOD1, OMIM number 147450), which binds copper and zinc; Fe and Mn types (SOD2, OMIM number 147460), which bind either iron or manganese; and the Ni type (SOD3, OMIM number 185490), which binds nickel (14). Mitochondrial SOD2 is an endogenous antioxidant enzyme that is significantly involved in limiting the oxidative burden (15). SOD2 converts superoxide into hydrogen peroxide and oxygen in the mitochondria and plays a major role in protecting cells from oxidative damage (16). A number of studies have demonstrated that the activity and expression of SOD2 changes significantly in patients with oral cancer, and either promotes or suppresses tumor formation (17–19). Therefore, in the present study it was hypothesized that the polymorphic variants of SOD2 may be involved in the development and prognosis of oral cancer. SOD1 is mainly located in the cytoplasm and accounts for ~85% of the total cellular SOD activity of most mammalian cells (20).

Polymorphic variants of SOD2 have been demonstrated to be factors that may enhance the risk of cancer development (21). The SOD2 SNP rs4880 (T>C) leads to a conformational change in the helical structure of the protein and has been associated with several carcinomas (9,15,22). rs5746136 in the 3′UTR of SOD2 is located 65 bp downstream of the poly A site. It is also <1 kb upstream from SP1 and the NF-κB transcription element sequences (23). To date, studies have rarely been conducted on polymorphisms of CuZnSOD in oral cancer (24). Therefore, in the present study, two other SNPs [rs1804450, Thr40Ile(C/T) and rs11556620, Asn87Ser(A/G)] were selected for investigation of their association with OSCC. A hospital-based case-control study was conducted to examine whether polymorphisms in rs4880, rs5746136, rs1804450 and rs11556620 SNPs are associated with OSCC. This is a novel study in which variations in SOD are evaluated with regard to OSCC risk.

## Material and methods

### Patients and controls

Han Chinese with clinically diagnosed OSCC (n=362) were recruited at the West China Hospital of Stomatology, Sichuan University (Chengdu, China), from December 2009 to March 2011. Han Chinese healthy individuals (n=358) who visited the general health check-up division of the West China Hospital of Stomatology, Sichuan University were recruited as controls.

All of the participants were residents of Sichuan Province, China. All subjects were informed of the detailed study protocol, signed consent forms and were asked to fill out a standardized questionnaire with two trained interviewers. The information required in the questionnaire included: i) Demographic factors, such as age, gender and ethnicity; ii) smoking and alcohol drinking habits (participants were considered as smokers if they had smoked at least one cigarette per day for the past six months or longer and as drinkers if they had drunk 100 ml of alcohol at least twice a week for the past six months); iii) disease, which consisted of systemic diseases or cancer in other sites; and iv) family history of cancer in parents, brothers and sisters. According to the inclusion criteria, participants with systemic diseases and/or a family history of cancer were filtered out by the standardized questionnaire and their medical records, so none of the participants in the study had a disease (except OSCC in the case group), a family history of cancer or the habit of betel nut chewing (there is no habit of betel nut chewing in Sichuan Province). All subjects in the patient group had their disease histopathologically confirmed and were previously untreated patients in the surgery departments. The OSCC information for all patients was collected from their medical records following their surgeries. Ethical approval was granted by the West China Hospital of Stomatology, Sichuan University ethics committee and all patients gave written consent prior to collection of the samples. Characteristics of the case group and control group are presented in Table I and the clinicopathological features of the case group are listed in Table II.

### DNA extraction and genotyping

Blood was taken from all participants by peripheral antecubital venous puncture and was stored at −20°C until analysis.

Genomic DNA was extracted using a salting-out method (25). The DNA concentration of each individual sample was measured on a NanoDrop 1000 Spectrophotometer using NanoDrop software, version 2.4.7c (NanoDrop Technologies Inc., Wilmington, DE, USA). The SOD2 and SOD1 genotypes rs4880, rs5746136, rs1804450 and rs11556620 were identified using a polymerase chain reaction-restriction fragment length polymorphism method (PCR-RFLP). The PCR primers were designed according to the reference sequence in the SNP database (dbSNP) of the NCBI (see Table III for primer sequences and reaction conditions). The PCR reaction consisted of 2.5 μl 10X PCR buffer, 0.1 mM dNTPs, 1 mM MgCl_2_, 2 units Taq polymerase (Tiangen, Beijing, China), 10 ng genomic DNA, 0.4 pM each primer and enough ddH_2_O to make the volume up to 25 μl per reaction.

The four PCR products were digested with 3 units of one of the specific endonucleases, *Taq*I, *Bsa*WI, *Acu*I or *Bts*I, for 4 h at 60°C or 37°C (Table III). PCR and digestion products were analyzed by electrophoresis in 7% or 8% polyacrylamide gels stained with silver nitrate. To confirm the genotyping results of each genetic polymorphism, 10–15% of the samples in each genotype group were randomly selected for Sanger sequencing, which was performed by Sangon Biotech (Shanghai, China). The results were 100% concordant with the RFLP analysis.

### Statistical analysis

The differences in the demographic characteristics were compared between the OSCC cases and controls using Student’s t-test and χ^2^ test. Student’s t-test was used to compare the difference in age and χ^2^ test was used to compare the differences in gender and tobacco and alcohol consumption. Models were run under the assumption of additive (AA vs. Aa vs. aa), dominant (AA vs. Aa/aa) or recessive (AA/Aa vs. aa) inheritance. Genotype frequencies of rs4880 and rs5746136 were compared between the OSCC cases and controls using the unconditional logistic regression. The odds ratios (ORs) with their 95% confidence intervals (CIs) were estimated by unconditional logistic regression. The adjusted P-values (Pas) were estimated by multiple logistic regression. The adjusted ORs (AORs), with their 95% CIs, of the association between the genotype frequencies and OSCC susceptibility were estimated by multiple logistic regression models after controlling for other covariates such as age, gender, smoking and drinking. The Hardy-Weinberg equilibrium was tested with a goodness of fit χ^2^ test with one degree of freedom to compare the observed genotype frequencies among the subjects with the expected genotype frequencies calculated byPowerStats v1.2 software (Promega Corporation, Madison, WI, USA). Statistical significance was assumed at P<0.05. SPSS Software, version 11.5 (SPSS, Inc., Chicago, IL, USA) was used for all of the statistical analyses. SHEsis software (http://analysis.bio-x.cn/SHEsisMain.htm) was applied for analysis of linkage disequilibrium (LD) (11).

## Results

The statistical data indicated there were no significant differences in age, gender and drinking between the patient group and the control group (P>0.05), but did identify that there were significant differences in smoking (P<0.0001; Table I).

Multiple logistic regression analyses were conducted for rs4880 (additive, CT vs. TT and CC vs. TT; dominant, CT/CC vs. TT; and recessive, CC vs. TT/CT) and rs5746136 (additive, AG vs. GG and AA vs. GG; dominant, AG/AA vs. GG; and recessive, AA vs. GG/GA). For SOD1, polymorphisms at rs1804450 and rs11556620 were not present in any of the OSCC and control subjects, hence these two SNPs were ruled out from subsequent analyses. The AORs and the respective 95% CIs of the genotype distributions of rs4880 and rs5746136 associated with the susceptibility of OSCC between the case group and control group are presented in Table IV. There was no significant association between genetic polymorphisms of rs5746136 and OSCC in the present study under additive, dominant and recessive models (Table IV). However, there was a significant 1.559-fold difference in the frequencies of the rs4880 genotypes containing the C allele between patients and control subjects in the dominant model (CC/CT vs. TT, 95% CI, 1.067–2.278). Moreover, the CT genotype was associated with a 1.484-fold significantly higher risk (CT vs. TT, 95% CI, 1.009–2.182) in the additive model. The genotype frequencies of rs4880 were significantly associated with OSCC in males in the additive and dominant models (Table V). In the additive model, the CT genotype was associated with a 1.663-fold significantly higher risk (CT vs. TT, 95% CI, 1.010–2.739). In the dominant model, the frequencies of the genotypes containing the C allele had a significant 1.809-fold difference (CC/CT vs. TT, 95% CI, 1.106–2.960) in males of the OSCC case group compared with the control group (Table V). The data indicate no significant association between genetic polymorphisms of rs5746136 with OSCC among individuals of either gender, nor of rs4880 with OSCC in females.

Furthermore, the Pa, AOR and 95% CI of genotypic frequencies and OSCC were also estimated among individuals with exposure or non-exposure to tobacco consumption. The genotype frequencies of rs4880 were significantly different between the OSCC patients who smoked and the control participants who smoked in the additive model (CT vs. TT) and dominant model (CC/CT vs. TT; Table VI). In the additive model, the CT genotype was associated with a 2.325-fold greater risk (CT vs. TT, 95% CI, 1.330–4.064). In the dominant model, the frequencies of the genotypes containing the C allele were significantly higher (by 2.448-fold) in patients with OSCC who smoked compared with those in healthy controls who smoked (CC/CT vs. TT, 95% CI, 1.417–4.230). However, no association was identified between the genetic polymorphism of rs4880 and OSCC in non-smokers after adjustment for other covariates (Table VI). Moreover, according to multiple logistic regression models after controlling for relevant factors, no significant association was identified between the genetic polymorphisms of rs5746136 and OSCC, regardless of whether individuals consumed tobacco.

The LD values for rs4880 and rs5746136 were evaluated using SHEsis. No significant LD was observed for the two loci; the r^2^ value was 0.009 and the D′ value was 0.275. PowerStats software analysis revealed no large deviations (P>0.05) from the Hardy-Weinberg equilibrium for these two loci (rs4880, P=0.87515; rs574616, P=0.58383).

The statistical power in this study was >80%, with an estimated odds ratio of 2.0 (rs4880, 86%; rs5746136, 82.2%).

## Discussion

OSCC is the most common type of malignancy of the oral cavity (26). In the past 30 years, the 5-year survival rate for patients with OSCC has not been improved substantially (~50%) (27). To date, the most effective treatment for OSCC is extensive surgical resection, with radiotherapy and/or chemotherapy prior to or following surgery (28,29). However, patients are frequently diagnosed at late stages. Gene therapy is also attracting more attention from an increasing number of researchers. The present study attempted to discover suitable novel biomarkers or genes which reflect the physiological state and changes of cells prior to or during the disease process to offer early and accurate prediction and diagnosis for patients with OSCC, particularly in early-stage OSCC (30).

The global prevalence of chronic diseases such as cancer, diabetes mellitus, hypertension, atherosclerosis and Alzheimer’s disease is increasing year on year. A number of these diseases are associated with oxidative stress (19) and for numerous cancers, oxidative stress is significantly involved. ROS influence apoptosis, tumor proliferation or promotion (16,31). Of the antioxidative enzymes, SOD2 is essential in the defense against mitochondrial superoxide radicals (32). Successive action of SOD2 detoxifies ROS (33). SOD2 dismutates the superoxide anion into one oxygen molecule and one hydrogen peroxide molecule, which is detoxified into water by glutathione peroxidase (34). In a previous study, intracellular ROS produced by the knockdown of SOD2 enhanced HIF-1α, which is a central regulator that controls the hypoxic response of mammalian cells through the induction of various target genes. Its expression contributes to the development and malignant progression of OSCC (35). In another study, the immunohistochemical staining intensities of SOD2 protein were demonstrated to be higher in well-differentiated OSCCs than in their normal cells of origin (normal oral squamous cells from the basal and spinous layer of oral mucosal tissue) by the examination of oral squamous cell carcinoma biopsies (19). Thus, the published studies suggest that elevated tumor SOD2 levels are associated with enhanced invasiveness of oral cancer (19). Few studies on SOD2 SNPs have been reported in association with oral cancer. Hakenewerth *et al* suggested that the SOD2 rs4342445 A allele was associated with 30% greater odds for oral cavity tumors in European- and African-American subjects (24). The SOD2 SNP rs4880 (T>C) in the gene sequence leads to a conformational change in the helical structure of the protein (36). The enzyme is less efficient for transport into mitochondria than the Ala allele (23). This allows Ala-SOD2 to be more efficiently imported into the mitochondrial matrix and to generate more active SOD2 compared with the Val variant (22). Carriage of one or two Ala-SOD2 alleles is reportedly associated with a greater risk of cancer. In a previous study, the Ala variant of SOD2 increased the risk of gastric cancer by altering the activity or content of SOD2 to lower its ability to detoxify ROS (22). A study has suggested that higher antioxidant activity due to an Ala variant of rs4880 in SOD2 may cause poorer survival following cyclophosphamide-containing breast cancer chemotherapy (37). In another study, patients with breast cancer and the high-activity genotype Ala/Ala in the SOD2 rs4880 SNP who were treated with regimens containing cyclophosphamide, but not with hormonal regimens, had significantly poorer prospects for survival than patients with low activity Val alleles (15). Yi *et al* reported that the SOD1-7958A/- genotype and -16Ala/- genotype, alcohol consumption, a family history of gastric cancer and *Helicobacter pylori* infection may all be risk factors for gastric cancer in the Han Chinese population (22). Xu *et al* hypothesized that people who carry the SOD2 rs4880 Ala allele may have active catalysis, resulting in the accumulation of hydrogen peroxide and secondary ROS generation (38).

In the present study, the common SNP for SOD2, rs5746136 C/G, was selected, as well as two other SNPs (rs1804450 and rs11556620 in SOD1) for evaluation of their association with susceptibility to OSCC in comparison with controls. The selection criteria for these three SNPs were that they are located in the coding region of SOD1, and their single-nucleotide changes would lead to an amino acid substitution. To the best of our knowledge, this is the first study to explore the polymorphisms of rs1804450 Thr/Ile and rs11556620 Asn/Ser that do not have genotype data available from the dbSNP of the NCBI. However, neither of these polymorphisms was identified in the members of the Sichuan Province Han population included in the present study.

In this study, no significant difference was observed in the genotype and allele frequencies of rs5746136 SNPs between the patients and controls. However, the C content of rs4880 was significantly higher in OSCC patients, particularly in the OSCC patients who smoked and in male OSCC patients compared with the control group. The C allele frequency of rs4880 was significantly higher in OSCC patients compared with normal subjects (CC/CT vs. TT, AOR=1.559, 95% CI, 1.067–2.278). The C allele frequency was also associated with a significantly higher risk of OSCC compared with the TT genotype in smokers (CC/CT vs. TT, AOR=2.448, 95% CI, 1.417–4.230). Cigarette smoke is one of the most important free radical-generating carcinogens. It was hypothesized that smoking and more highly active SOD2 (C allele) may lead to the accumulation of higher levels of hydrogen peroxide and secondary ROS generation, which increases the risk of DNA damage and OSCC. Nevertheless, whether intracellular ROS levels are changed in parallel with the SOD2 rs4880 genotypes in the OSCC microenvironment requires further investigation. The results of the present study corresponded well with other studies on tumors, such as adult brain tumors (OR=2.0; 95% CI, 1.0–4.2) (9), prostate cancer (OR=1.44; 95% CI, 1.1–1.9) (39), gastric cancer (OR=2.04; 95% CI, 1.19–3.49) (22) and breast cancer (40).

In conclusion, the present study provides the first evidence that a SOD2 polymorphism (C allele of rs4880) may convey a genetic susceptibility to OSCC in a Han Chinese population, but the rs5746136 polymorphism of SOD2 and the SOD1 polymorphisms did not. These results were consistent with previous studies of other types of cancer. This suggests that rs4880 of SOD2 may be a potential marker for disease diagnosis, further supporting the hypothesis that SOD2 is important in carcinogenesis. Further studies with larger samples in diverse ethnic populations are required to verify these conclusions.

## Figures and Tables

**Table I:** Characteristics of the case and control groups.

Characteristics	Case (n=362)	Control (n=358)	P-value^a^
Age (years)			
Range	19–87	28–81	
Mean + SD	60.89±11.57	59.98±11.41	0.786
Gender, n (%)			
Males	227 (62.7)	205 (57.3)	
Females	135 (37.3)	153 (42.7)	0.136
Smoking, n (%)			
Yes	254 (70.2)	158 (44.1)	
No	108 (29.8)	200 (55.9)	<0.0001^b^
Drinking, n (%)			
Yes	208 (57.5)	184 (51.4)	
No	154 (42.5)	174 (48.6)	0.102

a Student’s t-test or χ^2^ test,

b P<0.05.

**Table II:** Clinicopathological features of the case group (n=362).

Characteristics	n	%
Site		
Tongue	124	34.3
Buccal mucosa	99	27.3
Gingiva	63	17.4
Floor of mouth	41	11.3
Palate	22	6.1
Lip	11	3.0
Maxillary sinus	2	0.6
Tumor Size		
T1 + T2	208	57.5
T3 + T4	154	42.5
Lymph node metastases		
N0	233	64.4
N+	129	35.6
Clinical stage		
I	58	16.0
II	101	27.9
III	71	19.6
IV	132	36.5
Pathological stage		
SCC I (highly differentiated)	253	69.9
SCC II (moderately differentiated)	99	27.3
SCC III (poorly differentiated)	10	2.8

SCC, squamous cell carcinoma.

**Table III:** PCR primers and restriction enzymes used for amplification of the corresponding fragments.

PCR products	SNP (rs#)	Polymorphism	Primer sequence	Annealing temp (°C)	Amplicon size (bp)	Restriction enzyme	Restriction products (bp)
SOD2	rs4880	Val16Ala(T/C)	GCTGTGCTTTCTCGTCTTCAG TGGTACTTCTCCTCGGTGACG	58	208	*Bsa*W1	41/167 or 208
SOD2	rs5746136	(C/T)	AAAAACCACTGGGTGACATCTAC AAGGGAAACACTCGGCTTTCT	60	94	*Taq*I	42/52 or 94
SOD1	rs1804450	Thr40Ile(C/T)	CACAACACCCACCTGCTGTATTA GTGAAGGTGTGGGGAAGCATT	57	94	*Acu*I	41/53 or 94
SOD1	rs11556620	Asn87Ser(A/G)	TTAGTGGCATCAGCCCTAATCCAT ATAGACACATCGGCCACACCA	60	105	*Bts*I	36/69 or 105

PCR, polymerase chain reaction; SOD, superoxide dismutase.

**Table IV:** Comparison of the genotype frequencies of the SOD 2 polymorphisms between OSCC patients and controls.

SNP	Model/Allele	Genotype	Case (%) n=362	Control (%) n=358	P-value^a^	OR^b^ (95% CI)	Pa^c^	AOR^d^ (95% CI)
rs4880	Additive	TT	272 (75.1)	296 (82.7)		1.00 (Ref)		
		CT	83 (23.0)	61 (17.0)	0.037	1.481 (1.024–2.142)	0.045^e^	1.484 (1.009–2.182)
		CC	7 (1.9)	1 (0.3)	0.033	7.618 (0.931–62.316)	0.121	5.388 (0.642–45.216)
	Dominant	TT	272 (75.1)	296 (82.7)		1.00 (Ref)		
		CT/CC	90 (24.9)	62 (17.3)	0.013	1.580 (1.099–2.271)	0.022^e^	1.559 (1.067–2.278)
	Recessive	TT/CT	355 (98.1)	357 (99.7)				
		CC	7 (1.9)	1 (0.3)	0.069	7.039 (0.862–57.510)	0.138	4.988 (0.595–41.803)
rs5746136	Additive	GG	99 (27.3)	103 (28.8)		1.00 (Ref)		
		AG	172 (47.5)	180 (50.3)	0.974	0.994 (0.703–1.405)	0.960	1.009 (0.703–1.449)
		AA	91 (25.1)	75 (20.9)	0.267	1.262 (0.836–1.905)	0.418	1.194 (0.778–1.833)
	Dominant	GG	99 (27.3)	103 (28.8)		1.00 (Ref)		
		AG/AA	263 (72.7)	255 (71.2)	0.671	1.073 (0.775–1.148)	0.715	1.065 (0.759–1.496)
	Recessive	GG/AG	271 (74.9)	283 (79.1)		1.00 (Ref)		
		AA	91 (25.1)	75 (20.9)	0.182	1.267 (0.895–1.795)	0.354	1.187 (0.826–1.839)

a P-values were estimated by unconditional logistic regression.

b The odds ratios (ORs) with their 95% confidence intervals (CIs) were estimated by unconditional logistic regression.

c Adjusted P-values (Pas) were estimated by multiple logistic regression after controlling for age, gender, smoking and drinking.

d The adjusted ORs (AORs) with their 95% CIs were estimated by multiple logistic regression models after controlling for age, gender, smoking and drinking.

e P<0.05.

SOD, superoxide dismutase; OSSC, oral squamous cell carcinoma, SNP, single nucleotide polymorphism; Ref, reference.

**Table V:** Comparison of the genotype frequencies of the SOD 2 polymorphisms among males and females.

A. Among males (case, n=227 and control, n=205)								

SNP	Model/Allele	Genotype	Case (%)	Control (%)	P-value^a^	OR (95% CI)^b^	Pa^c^	AOR (95% CI)^d^
rs4880	Additive	TT	166 (73.1)	170 (82.9)		1.00 (Ref)		
		CT	56 (24.7)	35 (17.1)	0.040	1.639 (1.021–2.631)	0.045^e^	1.663 (1.010–2.739)
		CC	5 (2.2)	0 (0)	-	-	-	-
	Dominant	TT	166 (73.1)	170 (82.9)		1.00 (Ref)		
		CT/CC	61 (26.9)	35 (17.1)	0.014	1.785 (1.118–2.848)	0.018^e^	1.809 (1.106–2.960)
	Recessive	TT/CT	222 (97.8)	205 (100)		1.00 (Ref)		
		CC	5 (2.2)	0 (0)	-	-	-	-
rs5746136	Additive	GG	64 (28.2)	58 (28.3)		1.00 (Ref)		
		AG	103 (45.4)	99 (48.3)	0.798	0.943 (0.601–1.478)	0.944	1.020 (0.594–1.751)
		AA	60 (26.4)	48 (23.4)	0.638	1.133 (0.674–1.905)	0.601	0.882 (0.550–1.413)
	Dominant	GG	64 (28.2)	58 (28.3)		1.00 (Ref)		
		AG/AA	163 (71.8)	147 (71.7)	0.982	1.005 (0.661–1.529)	0.739	0.928 (0.598–1.440)
	Recessive	GG/AG	167 (73.6)	157 (76.8)		1.00 (Ref)		
		AA	60 (26.4)	48 (23.4)	0.470	1.175 (0.759–1.820)	0.672	1.103 (0.701–1.735)

B. Among females (case, n=135 and control, n=153)								

SNP	Model/Allele	Genotype	Case (%)	Control (%)	P-value^a^	OR (95% CI)^b^	Pa^c^	AOR (95% CI)^d^

rs4880	Additive	TT	106 (78.5)	126 (82.3)		1.00 (Ref)		
		CT	27 (20.0)	26 (17.0)	0.489	1.234 (0.679–2.243)	0.535	1.227 (0.642–2.344)
		CC	2 (1.5)	1 (0.7)	0.595	2.377 (0.213–26.584)	0.660	1.759 (0.142–21.738)
	Dominant	TT	106 (78.5)	126 (82.3)		1.00 (Ref)		
		CT/CC	29 (21.5)	27 (17.7)	0.412	1.277 (0.712–2.290)	0.488	1.251 (0.664–2.355)
	Recessive	TT/CT	133 (98.5)	152 (99.3)		1.00 (Ref)		
		CC	2 (1.5)	1 (0.7)	0.502 (0.205–25.493)	2.286	0.682 (0.137–20.834)	1.690
rs5746136	Additive	GG	35 (25.9)	45 (29.4)		1.00 (Ref)		
		AG	69 (51.1)	81 (53.0)	0.744 (0.634–1.891)	1.095	0.415 (0.703–2.348)	1.285
		AA	31 (23.0)	27 (17.6)	0.260 (0.748–2.911)	1.476	0.159 (0.811–3.580)	1.704
	Dominant	GG	35 (25.2)	45 (29.4)		1.00 (Ref)		
		AG/AA	100 (74.8)	108 (70.6)	0.510 (0.709–2.000)	1.190	0.257 (0.786–2.469)	1.393
	Recessive	GG/AG	104 (77.2)	126 (82.4)		1.00 (Ref)		
		AA	31 (22.8)	27 (17.6)	0.262	1.391 (0.781–2.478)	0.248	1.445 (0.773–2.702)

a P-values were estimated by unconditional logistic regression.

b The odds ratios (ORs) with their 95% confidence intervals (CIs) were estimated by unconditional logistic regression.

c Adjusted P-values (Pas) were estimated by multiple logistic regression after controlling for age, gender, smoking and drinking.

d The adjusted ORs (AORs) with their 95% CIs were estimated by multiple logistic regression models after controlling for age, smoking and drinking.

e P<0.05.

SOD, superoxide dismutase; OSSC, oral squamous cell carcinoma; SNP, single nucleotide polymorphism; Ref, reference.

**Table VI:** Comparison of the genotype frequencies of the SOD 2 polymorphisms among smokers and non-smokers.

A. Among smokers (case, n=254 and control, n=158)								

SNP	Model/Allele	Genotype	Case (%)	Control (%)	P-value^a^	OR (95% CI)^b^	Pa^c^	AOR (95% CI)^d^
rs4880	Additive	TT	185 (72.8)	135 (85.4)		1.00 (Ref)		
		CT	63 (24.8)	22 (13.9)	0.006	2.090 (1.225–3.563)	0.003^e^	2.325 (1.330–4.064)
		CC	6 (2.4)	1 (0.7)	0.138	4.378 (0.521–36.792)	0.134	5.404 (0.594–49.189)
	Dominant	TT	185 (72.8)	135 (85.4)		1.00 (Ref)		
		CT/CC	69 (27.2)	23 (14.6)	0.003	2.189 (1.300–3.688)	0.001^e^	2.448 (1.417–4.230)
	Recessive	TT/CT	248 (97.6)	157 (99.3)		1.00 (Ref)		
		CC	6 (2.4)	1 (0.7)	0.187	3.789 (0.453–31.849)	0.181	4.485 (0.498–40.420)
rs5746136	Additive	GG	68 (26.8)	47 (29.7)		1.00 (Ref)		
		AG	121 (47.6)	70 (44.3)	0.462	1.195 (0.744–1.920)	0.335	1.272 (0.780–2.074)
		AA	65 (25.6)	41 (25.9)	0.740	1.096 (0.639–1.879)	0.606	1.157 (0.665–2.013)
	Dominant	GG	68 (26.8)	47 (29.7)		1.00 (Ref)		
		AG/AA	186 (73.2)	111 (70.3)	0.513	1.158 (0.746–1.798)	0.372	1.229 (0.782–1.932)
	Recessive	GG/AG	189 (74.4)	117 (74.1)		1.00 (Ref)		
		AA	65 (25.6)	41 (25.9)	0.935	0.981 (0.623–1.545)	0.993	0.998 (0.626–1.592)

B. Among non-smokers (case, n=108 and control, n=200)								

SNP	Model/Allele	Genotype	Case (%)	Control (%)	P-value^a^	OR (95% CI)^b^	Pa^c^	AOR (95% CI)^d^

rs4880	Additive	TT	87 (80.6)	161 (80.5)		1.00 (Ref)		
		CT	20 (18.5)	39 (19.5)	0.864	0.949 (0.521–1.727)	0.962	1.016 (0.540–1.911)
		CC	1 (0.9)	0 (0)	-	-	-	-
	Dominant	TT	87 (80.6)	161 (80.5)		1.00 (Ref)		
		CT/CC	21 (19.4)	39 (19.5)	0.991	0.996 (0.552–1.800)	0.848	1.064 (0.569–1.987)
	Recessive	TT/CT	107 (99.1)	200 (100)		1.00 (Ref)		
		CC	1 (0.9)	0 (0)	-	-	-	-
rs5746136	Additive	GG	31 (28.7)	56 (28.0)		1.00 (Ref)		
		AG	51 (47.2)	110 (55.0)	0.527	0.838 (0.483–1.452)	0.462	0.804 (0.448–1.440)
		AA	26 (24.1)	34 (17.0)	0.346	1.381 (0.705–2.708)	0.303	1.457 (0.712–2.978)
	Dominant	GG	31 (28.7)	56 (28.0)		1.00 (Ref)		
		AG/AA	77 (71.3)	144 (72.0)	0.896	0.966 (0.575–1.623)	0.862	0.952 (0.550–1.649)
	Recessive	GG/AG	82 (75.9)	166 (83.0)		1.00 (Ref)		
		AA	26 (24.1)	34 (17.0)	0.135	1.548 (0.871–2.751)	0.098	1.676 (0.909–3.091)

a P-values were estimated by unconditional logistic regression.

b The odds ratios (ORs) with their 95% confidence intervals (CIs) were estimated by unconditional logistic regression.

c Adjusted P-values (Pas) were estimated by multiple logistic regression after controlling for age, gender, smoking and drinking.

d The adjusted ORs (AORs) with their 95% CIs were estimated by multiple logistic regression models after controlling for age, gender and drinking.

e P<0.05.

SOD, superoxide dismutase; OSSC, oral squamous cell carcinoma; SNP, single nucleotide polymorphism; Ref, reference.
